# Multi-indicator comparative evaluation for deep learning-based protein sequence design methods

**DOI:** 10.1093/bioinformatics/btae037

**Published:** 2024-01-23

**Authors:** Jinyu Yu, Junxi Mu, Ting Wei, Hai-Feng Chen

**Affiliations:** State Key Laboratory of Microbial Metabolism, Joint International Research Laboratory of Metabolic & Developmental Sciences, Department of Bioinformatics and Biostatistics, National Experimental Teaching Center for Life Sciences and Biotechnology, School of Life Sciences and Biotechnology, Shanghai Center for Systems Biomedicine, Shanghai Jiao Tong University, Shanghai 200240, China; State Key Laboratory of Microbial Metabolism, Joint International Research Laboratory of Metabolic & Developmental Sciences, Department of Bioinformatics and Biostatistics, National Experimental Teaching Center for Life Sciences and Biotechnology, School of Life Sciences and Biotechnology, Shanghai Center for Systems Biomedicine, Shanghai Jiao Tong University, Shanghai 200240, China; State Key Laboratory of Microbial Metabolism, Joint International Research Laboratory of Metabolic & Developmental Sciences, Department of Bioinformatics and Biostatistics, National Experimental Teaching Center for Life Sciences and Biotechnology, School of Life Sciences and Biotechnology, Shanghai Center for Systems Biomedicine, Shanghai Jiao Tong University, Shanghai 200240, China; State Key Laboratory of Microbial Metabolism, Joint International Research Laboratory of Metabolic & Developmental Sciences, Department of Bioinformatics and Biostatistics, National Experimental Teaching Center for Life Sciences and Biotechnology, School of Life Sciences and Biotechnology, Shanghai Center for Systems Biomedicine, Shanghai Jiao Tong University, Shanghai 200240, China

## Abstract

**Motivation:**

Proteins found in nature represent only a fraction of the vast space of possible proteins. Protein design presents an opportunity to explore and expand this protein landscape. Within protein design, protein sequence design plays a crucial role, and numerous successful methods have been developed. Notably, deep learning-based protein sequence design methods have experienced significant advancements in recent years. However, a comprehensive and systematic comparison and evaluation of these methods have been lacking, with indicators provided by different methods often inconsistent or lacking effectiveness.

**Results:**

To address this gap, we have designed a diverse set of indicators that cover several important aspects, including sequence recovery, diversity, root-mean-square deviation of protein structure, secondary structure, and the distribution of polar and nonpolar amino acids. In our evaluation, we have employed an improved weighted inferiority–superiority distance method to comprehensively assess the performance of eight widely used deep learning-based protein sequence design methods. Our evaluation not only provides rankings of these methods but also offers optimization suggestions by analyzing the strengths and weaknesses of each method. Furthermore, we have developed a method to select the best temperature parameter and proposed solutions for the common issue of designing sequences with consecutive repetitive amino acids, which is often encountered in protein design methods. These findings can greatly assist users in selecting suitable protein sequence design methods. Overall, our work contributes to the field of protein sequence design by providing a comprehensive evaluation system and optimization suggestions for different methods.

## 1 Introduction

Proteins are vital components in biological systems, performing a diverse range of functions. However, the natural protein sequences sampled so far represent only a small fraction of the immense number of possible protein sequences. Even for a relatively short protein of 100 residues, there are about 10^130^ possible amino acid sequences, surpassing the collection of different proteins found in all known species (Baker 2019). To leverage the untapped potential of protein structures, it is desirable to optimize existing applications and design proteins with new functions. Protein design can be broadly categorized into two types: protein backbone design and fixed-backbone protein sequence design (Korendovych and DeGrado 2020). In particular, fixed-backbone protein sequence design aims to assign appropriate amino acid types to each residue, enabling the designed protein to fold into a desired backbone structure.

The earliest protein design algorithms relied on classical physical principles, with computational protein design (CPD) emerging as the most widely used approach (Woolfson 2021). One highly successful CPD method is Rosetta (Liu and Kuhlman 2006), a suite of software tools for protein structure prediction and design. Rosetta stands out for its computational accuracy, scalability, and technical customization capabilities. Moreover, Rosetta can incorporate experimental data to improve the accuracy and reliability of the designed protein structures.

In recent years, a set of machine learning methods has been developed (Ferruz *et al.* 2022), offering new possibilities for *de novo* protein design. These methods are more efficient and accurate compared to physics-based approaches, making them the mainstream choice for protein sequence design. Many machine learning-based methods adopt a concurrent strategy (Huang *et al.* 2023), such as ProteinMPNN (Dauparas *et al.* 2022) and ESM-IF1 (Hsu *et al.* 2022), where the amino acid types for all residues in the protein are determined concurrently during the design process. While concurrent strategies demonstrate high inference efficiency, they may not generalize well to proteins outside the training set. Alternatively, some methods employ an iterative strategy, where residues at target positions are iteratively mutated to improve the overall fitness between the protein sequence and the target structure. Although this approach yields better results, it often sacrifices efficiency. Furthermore, machine learning-based protein sequence design methods vary significantly in terms of feature extraction, model architecture, and other specific details, resulting in considerable variability in their performance outcomes.

Currently, there is no definitive and accurate way to evaluate different protein sequence design methods, making it challenging to directly assess the quality of designed sequences. Commonly used evaluation metrics in deep learning, such as the area under the curve (AUC), often fail to capture the biological significance that holds greater importance (Castorina *et al.* 2023). Therefore, it is necessary to employ more appropriate indicators for a comprehensive evaluation of different methods. Existing fixed-backbone sequence design methods primarily focus on sequence recovery (Qi and Zhang 2020), where the designed sequences are evaluated based on their identity to native sequences using the backbone of a natural protein as a reference. However, this approach has limitations, particularly an excessive focus on recovery while neglecting sequence diversity. Ideally, designed sequences should span a wide range of protein sequence space and exhibit a high degree of diversity. Although some protein sequence design methods incorporate adjustable diversity parameters or use indicators like the TM-score to evaluate the results (Liu *et al.* 2022), systematic comparisons between these methods are lacking due to varying evaluation approaches.

Our work aims to establish a multi-indicator evaluation model to address the question of which protein sequence design methods are more applicable and under what circumstances. This evaluation model can assist developers in enhancing the comprehensive capabilities of their methods and provide insights for optimization. Additionally, our model can help identify sequences with a higher likelihood of successful folding, thereby increasing the success rate of experimental verification and reducing associated costs. We have designed evaluation indicators from different perspectives, including protein sequence diversity, protein sequence foldability, and protein structure rationality. We evaluate several widely used methods using protein datasets that are not part of these methods’ training and test sets to ensure unbiased evaluation. Subsequently, we statistically analyze the designed evaluation indicators and construct a comprehensive evaluation model based on these indicators. This evaluation model provides a more comprehensive and professional perspective for comparing different methods, enabling users to quickly identify suitable methods, while developers can gain insights for further development and optimization.

## 2 Materials and methods

### 2.1 Comparison models and datasets

We selected eight widely used protein design methods for a comprehensive evaluation (Table 1). To make the evaluation model more scientific, we introduced a noise method that consists of 3000 randomly generated sequences based on amino acid ratios in nature (UniProt Consortium 2019). Comparing its indicators with those of normal protein sequence design methods can reflect the performance of design indicators in practical applications, evaluate the usefulness of indicators, and provide ideas for adjusting and optimizing indicators. At the same time, the noise method will also be incorporated into our evaluation system to improve the performance of the evaluation model.

**Table 1.** Selected protein sequence design methods.

Method	Architecture	Concurrent	Time received	Experimental validations
Structured Transformer (Ingraham *et al.* 2019)	Modified Transformer	Yes	2019/12	None
ProteinSolver (Strokach *et al.* 2020)	GNN	Yes	2020/04	Secondary structure
3D CNN (Anand *et al.* 2022)	CNN	No	2021/08	Crystal structure
ABACUS-R (Liu *et al.* 2022)	Modified Transformer	No	2021/12	Crystal structure
ESM-IF1 (Hsu *et al.* 2022)	Modified Transformer	Yes	2022/07	None
ProtienMPNN (Dauparas *et al.* 2022)	Message passing neural network	Yes	2022/05	Crystal structure
GPD (Mu *et al.* 2023)	Modified Transformer (Ying *et al.* 2021)	Yes	2023/08	Enzyme activity
PiFold (Gao *et al.* 2022)	GNN	Yes	2022/09	None

In order to ensure fair evaluation, it is important to avoid using protein structures from the Class Architecture Topology Homologous (CATH) database, as most protein design models rely on this database for training and testing. To investigate the application performance of different models, we selected 14 known *de novo* proteins that do not exist in CATH database (Rocklin *et al.* 2017). *De novo* protein design represents a key application scenario for protein design (Korendovych and DeGrado 2020), and the exclusion of these proteins from the CATH database aligns with our research objectives. For each structure of these 14 known *de novo* proteins, we designed 100 sequences, resulting in a total of 14 × 100 sequences. All designs and evaluations were executed on CPU for consistency. We also randomly selected 103 single-chain proteins from the CATH database and 14 proteins were selected to ensure the diversity of structures as much as possible. Then we used the chosen protein design method to design them with the same standard as *de novo* proteins (Supplementary Table S1). After all, single-chain proteins also represent a key application scenario for protein design.

### 2.2 Evaluation indicators

After obtaining the corresponding sequences, we faced the challenge that many of the indicators we developed required the use of protein structures. To address this, we relied on single-chain protein structure prediction (folding) technology, which has seen remarkable advancements. Among them, AlphaFold2 (Bryant *et al.* 2022) and ESMFold (Lin *et al.* 2023) have the best prediction effect. We finally chose ESMFold for folding the designed sequences, because it is more accurate in predicting orphan proteins like the *de novo* proteins than AlphaFold2 which relies on Multiple Sequence Alignment (MSA) information (Meng *et al.* 2023).

**Figure 1.**
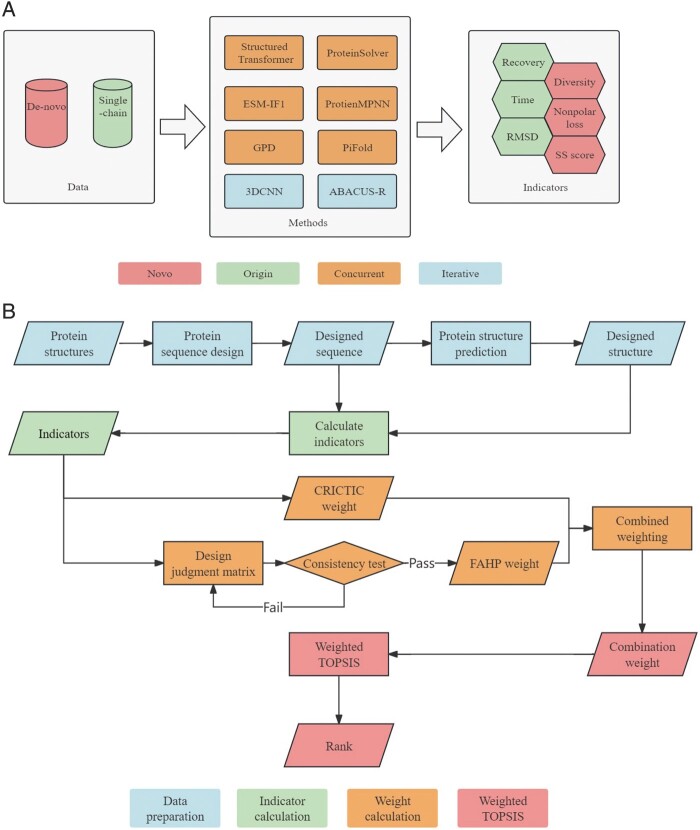
Pipeline of evaluation model. (**A**) Methods and data. Different types or different strategies are distinguished by different colors. (**B**) Flow chart of evaluation model calculation process. Different steps are distinguished by different colors.

The crux of our research lies in effectively utilizing the fundamental data, which includes factors such as the computational time, designed sequences, and folded structures, to derive indicators that reflect practical significance. To achieve this, we devised a range of indicators from various perspectives (see “Evaluation indicators” in Supplementary Text). These indicators encompass parameters such as Recovery, Diversity, Root-Mean-Square Deviation (RMSD) of protein structures, secondary structure score (SS score), and nonpolar amino acid loss (Nonpolar loss). Through these indicators, we aim to provide a comprehensive evaluation of the performance and practical relevance of the protein design methods (Fig. 1A). Recovery is the similarity between the designed sequences and native sequences. Diversity will use Clustalw2 (Larkin *et al.* 2007) to compare the designed sequences in pairs, and count the average difference between the designed sequences according to the alignment results. RMSD is a quantitative and fundamental structural evaluation indicator that measures the disparities between two structures. And based on existing research (Wang *et al.* 2022), Nonpolar loss was designed to reflect the rationality of amino acid types in the structure. When there are more nonpolar amino acids on the surface, the Nonpolar loss will be higher, then we will compare the value of this indicator in the design structure and the native structure. For SS Score, we employed a secondary structure prediction tool to anticipate the secondary structure based on the sequence. And calculated the similarity between the predicted secondary structure of the generated sequences and the native sequence.

### 2.3 Evaluation method

The Analytic Hierarchy Process (AHP) and the Technique for Order Preference by Similarity to Ideal Solution (TOPSIS) are widely recognized and frequently employed multi-attribute decision-making (MADM) techniques. These methods are often combined (Kubler *et al.* 2016) to enhance the effectiveness of evaluations. Additionally, we introduced the CRiteria Importance Through Intercriteria Correlation (CRITIC) method to increase objectivity to our evaluation model and reduce the influence of the correlation between indicators. It can also be used in combination with TOPSIS to improve the accuracy of evaluation (Abdel-Basset and Mohamed 2020). In our evaluation system, we adopted the weighted TOPSIS method integrating CRITIC objective weight and AHP subjective weight as our evaluation technique (shown in Fig. 1). The entire process comprised four distinct steps: data preparation, indicator calculation, weight calculation, and weighted TOPSIS.

### 2.4 Data preprocessing

Since RMSD, Time, and Nonpolar loss are all cost attributes, that is, the larger the value means the worse the performance. We need to normalize these indicators. For cost attributes Time and Nonpolar loss, we used a conventional linear normalization transforming cost attributes to benefit attributes, that is, for a certain indicator j, the normalization value of method i is the maximum value of the indicator j minus the original indicator value of method i.

However, for the RMSD indicator, after debugging, we ultimately chose to normalize it by taking the reciprocal. Taking the reciprocal reduces the influence of outliers on the evaluation model. Additionally, when the RMSD is smaller, it can clearly differentiate between numerical differences. Although the differences become smaller when the RMSD value is larger, the model evaluation can still be accurate due to the higher weight given to the RMSD.

To eliminate dimension, we need to standardize the data. We found that using Z-score normalization for the evaluation model is more stable and reasonable than using Minmax normalization. This is due to the limitations of Minmax normalization in handling outliers. Therefore, we used Z-score normalization.

### 2.5 Weighting

After normalizing the indicators, we utilized the weighted TOPSIS method, allowing for adjustable comprehensive evaluations by incorporating various weights. However, a limitation arises when using TOPSIS for distance calculations, specifically when there is a linear correlation between attributes (Yue 2011). In such cases, principal component analysis (PCA) and factor analysis can be employed to extract and rotate the original indicators, reducing the impact of the correlation. Given the small number of indicators, we did not consider replacing the Euclidean distance with Mahalanobis distance as a solution. This is because utilizing Mahalanobis distance to eliminate correlation in the final distance calculation would also nullify the assigned weights, resulting in suboptimal results. Hence, we adopted the CRITIC weights to ensure a meaningful evaluation.

‘CRITIC Method’ is a comprehensive evaluation method based on the variability of evaluation indicators and the conflicts between indicators to measure the objective weights of the indicators (Diakoulaki *et al.* 1995).

In the CRITIC with a fixed standard deviation, smaller conflicts between indicators lead to smaller weights. Conversely, larger conflicts lead to larger weights. Furthermore, when the positive correlation between two indicators is stronger (correlation parameter closer to 1), the conflicts are smaller, indicating a greater similarity in the information reflected by these two indicators in the evaluation of the quality of the solutions. Therefore, the introduction of CRITIC weights can, to a certain extent, overcome the limitations of TOPSIS mentioned above when there is a correlation between the data. The objective weight $W^{1}$ of each indicator are obtained by CRITIC method (see Supplementary Text for calculation process).

‘AHP’ (Saaty 1990) is a broadly applied MADM method to determine the subjective weights of criteria and priorities of alternatives in a structured manner based on pairwise comparison. However, subjective judgments during comparison might be imprecise. In addition, when individuals are judging an event, even using the same words, they may significantly differ since each of them has different subjective perception or personality(Mardani *et al.* 2015).

To overcome this problem, fuzzy numbers were introduced in a way to help linguistic variables be expressed appropriately. As a popular methodology for handling imprecision, fuzzy sets proposed by Zadeh (1965) are combined with AHP, namely FAHP. This integrated method maintains the advantage of AHP and has been the most widely used fuzzy multiple criteria decision-making (FMCDM) method (Kubler *et al.* 2016). FMCDM is one of the most widely used decision methodologies in engineering, technology, science and management and business (Mardani *et al.* 2015). The procedure of building a FAHP model follows establishing the judgment matrix, measuring the consistency and defuzzifying the fuzzy weights. Various techniques exist for each aspect. For procedure of building a FAHP model, we chose the most suitable method according to the existing research (Liu *et al.* 2020).

After we obtain the FAHP subjective weight $W^{2}$ (see Supplementary Text for calculation process). The final weight W is the combination of objective weight $W^{1}$ and subjective weight $W^{2}$, where the proportions of $W^{1}$ and $W^{2}$ are 5:5.

‘TOPSIS’ is based on the principle of relative ranking (Tzeng and Huang 2011). It evaluates the overall distance of each solution in the system to the ideal best and worst solutions through certain calculations. If a solution is closer to the ideal best solution and further from the worst solution, we have reason to believe that this solution is better. Based on the calculated relative nearness $C_{i}$ (Supplementary Text), we can rank the solutions, where larger values indicate better design methods.

### 2.6 Temperature selection

As mentioned previously, several existing methods have focused on improving the diversity of output sequences. Methods like ProteinMPNN and ABACUS-R have introduced a temperature parameter to adjust the diversity of model output sequences. The original intention behind the parameter design was to allow users to adjust it according to their needs. However, these models typically select parameters with the highest recovery or the lowest difference as default values, assuming them to be the best temperature parameters. We believe that the temperature parameter offering the highest recovery might not necessarily be the most suitable one.

To address this concern, we conducted a statistical analysis of the design outcomes of ProteinMPNN and ABACUS-R using different temperature parameters. Moreover, we employed the McKinsey Selection Matrix, a renowned tool for managing complex business portfolios (Yang and Jiang 2018), to select an ideal temperature parameter. Traditionally, the McKinsey matrix evaluates business units based on industry attractiveness on the y-axis and the competitive strength of the units on the x-axis, providing a two-dimensional evaluation. In our case, we modified the McKinsey matrix to suit our specific problem. We placed recovery on the x-axis and diversity on the y-axis to construct the modified McKinsey matrix.

## 3 Results

### 3.1 Indicators and ranking results

**Figure 2.**
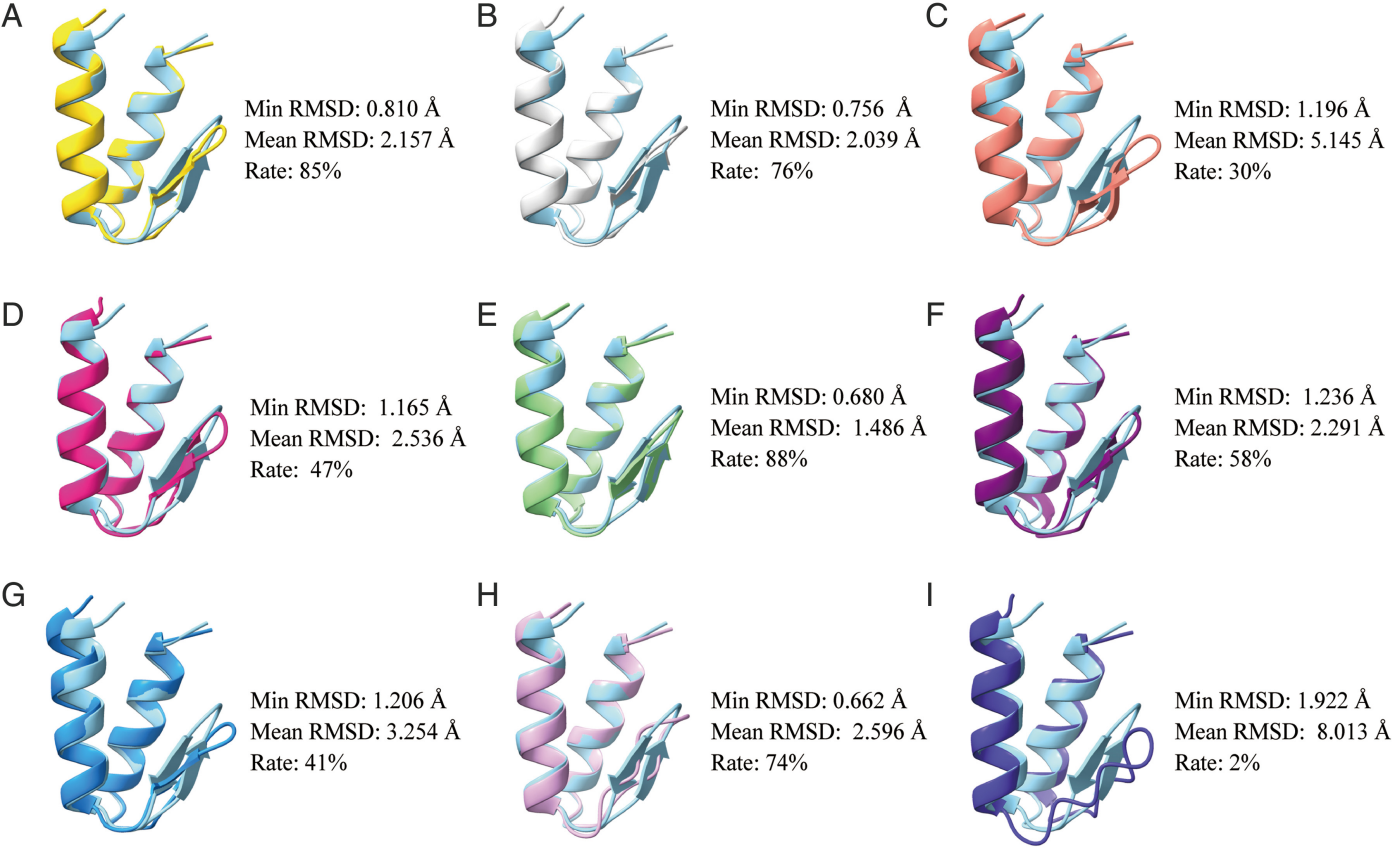
Design results of different methods. The alignment between native structure (light blue) and the best folding design sequence of HEEH_rd3_0223 by different methods. The minimum RMSD value (Min RMSD) and the average RMSD (Mean RMSD) and qualified ratio of 100 folded structures (Rate) are also shown, respectively. (**A**) ProteinMPNN (*T* = 0.1). (**B**) ESM-IF1. (**C**): ABACUS-R. (**D**) GPD. (**E**) 3D CNN (LogP). (**F**) PiFold. (**G**) Structured Transformer. (**H**) 3D CNN (Energy). (**I**) ProteinSolver.

Considering the design strategies for 3D Convolutional Neural Network (CNN), the optimal temperature parameter in ProteinMPNN, and the noise methods, a total of 11 *de novo* protein sequence design methods were employed to design and sample 14 *de novo* proteins. These sampled conformations were subsequently evaluated using the weighted TOPSIS evaluation model. The ranking results and design results can be found in Table 2 and Fig. 2.

**Table 2.** Indicators and ranking results for *de novo* proteins.

Method and weight	Recovery	Diversity	Time (s)	SS score	RMSD (Å)	Nonpolar loss	$C_{i}$	Rank
Structured Transformer	0.441	0.074	13	0.966	1.526	1.352	0.557	8
Protein Solver	0.246	0.186	180	0.792	5.354	1.389	0.394	10
3D CNN (Energy)	0.421	0.325	536544	0.931	2.130	1.085	0.539	9
3D CNN (LogP)	0.445	0.272	536544	0.952	1.620	1.027	0.592	6
ABAC US-R	0.457	0.124	233280	0.972	1.482	0.968	0.615	4
ESM-IF1	0.477	0.184	1980	0.965	1.265	1.201	0.724	3
ProteinMPNN (*T* = 0.1)	0.487	0.168	112	0.975	1.019	1.061	0.784	2
ProteinMPNN (*T* = 0.5)	0.430	0.299	112	0.965	1.320	1.174	0.792	1
GPD	0.462	0.219	35	0.967	1.758	1.333	0.611	5
PiFold	0.428	0.141	221	0.945	1.592	1.464	0.566	7
Noise	0.049	0.111	–	0.185	11.830	1.628	0.280	11
AHP weight	0.050	0.163	0.038	0.089	0.48	0.177		
CRITIC weight	0.100	0.196	0.367	0.099	0.104	0.135		
Final weight	0.075	0.179	0.202	0.094	0.293	0.156		

According to our evaluation model, ProteinMPNN and ESM-IF1 emerged as the best performing methods in *de novo* protein design. Specifically, the results of ProteinMPNN with an adjusted temperature parameter (*T* = 0.5) were particularly favorable, indicating that our temperature selection method and evaluation model are complementary and effective. ABACUS-R and the best LogP-based 3D CNN, both utilizing an iterative strategy, also achieved relatively high ranks. Despite the longer sequence generation time, these methods produced sequences with more reasonable structures, as evidenced by lower Nonpolar loss. On the other hand, ProteinSolver exhibited the poorest performance. It is noteworthy that newly released methods generally demonstrated better overall performance.

Although ProteinMPNN and ESM-IF1 exhibited good RMSD performance, their Nonpolar loss remained high. This suggests that these two indicators are not strongly correlated, potentially due to the excessive folding ability of current protein folding prediction methods. Consequently, sequences that lead to poor folding under natural conditions might be optimized into folded structures using protein folding prediction tools. This underscores the importance of not relying solely on RMSD as the criterion for judging structural excellence. Furthermore, the introduction of the noise method increased the stability and effectiveness of our evaluation across different methods (Supplementary Table S2).

After successfully establishing our evaluation model, we also attempted to apply it to assess the ability of each method to design single-chain proteins with a fixed backbone. To achieve this, certain optimizations were necessary, particularly in the statistical analysis of RMSD. As single-chain proteins are longer and pose greater design challenges, we improved our RMSD calculation method by dividing it into the ratio of qualified protein RMSD and the mean RMSD of qualified proteins. Our criterion for qualifying proteins was RMSD < 2 Å.

Due to time constraints, we focused on collecting data on the design performance of methods using concurrent design for 14 single-chain proteins. The results can be found in Table 3, with the AHP judgment matrix provided in Supplementary Table S3.

**Table 3.** Indicators and ranking results of single-chain proteins.^a^

Method and weight	Recovery	Diversity	Time (s)	SS score	Nonpolar loss	RMSD (Å)	Qualified rate	$C_{i}$	Rank
Structured Transformer	0.279	0.105	13	0.738	0.759	1.763	0.151	0.456	3
ProteinSolver	0.176	0.511	1980	0.632	1.157	1.827	0.001	0.386	5
ESM-IF1	0.320	0.277	112	0.735	0.805	1.534	0.248	0.642	2
ProteinMPNN	0.264	0.226	35	0.749	0.805	1.477	0.278	0.740	1
GPD	0.280	0.278	221	0.740	0.923	1.782	0.045	0.402	4
PiFold	0.256	0.114	180	0.680	1.074	1.707	0.076	0.361	6
AHP weight	0.036	0.104	0.028	0.062	0.112	0.329	0.329		
CRITIC weight	0.103	0.216	0.237	0.106	0.109	0.120	0.108		
Final weight	0.070	0.160	0.133	0.084	0.111	0.225	0.219		

a RMSD is the average value of qualified RMSD, and qualified rate is the ratio of qualified protein over all folded proteins.

By splitting the original structure-related indicator RMSD into two separate indicators, our evaluation model was able to provide a more comprehensive assessment of method performance. Consequently, methods that exhibited better sequence folding capabilities achieved higher rankings in the evaluation. As a result, ProteinMPNN consistently maintained its leading position, while Structured Transformer outperformed GPD and PiFold. It is worth noting that the performance of all methods deteriorated when designing longer single-chain proteins. The observed decrease in performance was not solely attributed to excessive RMSD values. In fact, we discovered that the diversity of qualified proteins was very similar to the overall diversity (RMSE = 0.0184). Thus, when RMSD was divided into two indicators, it was more appropriate to utilize the unadjusted diversity as a measure.

### 3.2 Temperature parameter evaluation

**Figure 3.**
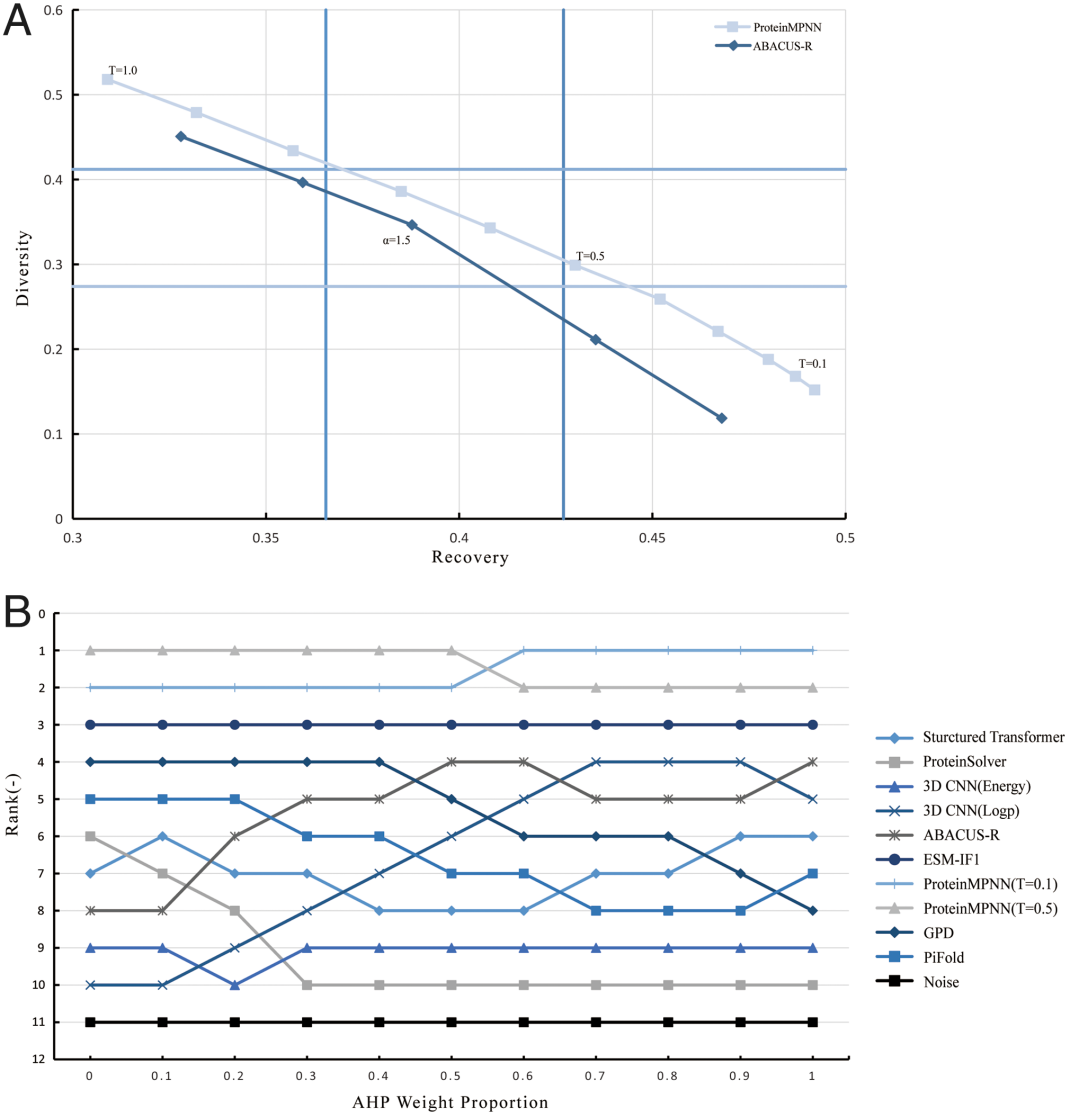
Temperature parameter selection results and sensitivity analysis. (**A**) Indicator changes caused by different temperature parameters. The red line represents the Recovery and Diversity Indicators of ProteinMPNN for the design results of 14 *de novo* proteins under 11 temperature parameters of *T* = 1.0, 0.9 … 0.2, 0.1, and 0.01. Where *T* = 0.1 is the default temperature parameter of ProteinMPNN. The blue line represents the statistical results of ABACUS-R under five temperature parameters of α = 0.8, 1, 1.5, 2, and 3.5, where the relationship between α and *T* is α = 1/*T*. α = inf is the default temperature parameter of ABACUS-R, and the statistical result is close to α = 3.5. Recovery threshold: *x* = 0.365, *x* = 0.427. Diversity threshold: *y* = 0.274, *y* = 0.412. (**B**) Sensitivity Analysis Change Chart. The ranking of 11 objects changes with the weight proportion of AHP. Object types are represented by different tags with different colors. AHP weight proportion = 0.6 means that the ratio of AHP weight to CRITIC weight is 6:4.

We made modifications to the McKinsey matrix to address our specific problem. We positioned Recovery on the X-axis and Diversity on the Y-axis, allowing us to construct the modified McKinsey matrix. To determine the boundaries of the nine boxes in the matrix, we utilized the K-means clustering algorithm. We clustered the selected indicators into three categories based on Recovery and Diversity. The average of the three cluster centers was then used as the threshold values. For Recovery, the threshold values were determined to be 3.655 and 4.27, while for Diversity, the thresholds were set at 2.74 and 4.12. By examining the McKinsey matrix and its thresholds, we observed that ProteinMPNN exhibited moderate diversity and excellent recovery at a temperature parameter of *T* = 0.5 (Fig. 3A). This suggests that using a temperature parameter of *T* = 0.5 could yield a greater number of designed sequences. Similarly, for ABACUS-R, α = 1.5 proved to be the preferable temperature parameter. Comparing the two methods, ProteinMPNN outperformed ABACUS-R in terms of both Recovery and Diversity across various temperature parameters. Analyzing the trend lines, it is evident that for ABACUS-R, higher temperature parameters corresponded to a lower Recovery loss at the expense of a higher Diversity improvement. Conversely, the performance of ProteinMPNN remained relatively consistent across different temperature parameters.

### 3.3 Model sensitivity analysis

Sensitivity analysis is a valuable technique that enables the assessment of how variations in input parameters affect model outputs. Its primary objectives are to evaluate the model’s stability, reliability, and response to uncertainty (Saltelli *et al.* 2008). Furthermore, sensitivity analysis can help identify which parameters exert a greater influence on the system or model.

In our protein design method evaluation model, we employed sensitivity analysis to evaluate and observe the impact of parameters on the model, providing insights into its stability. By adjusting the weight parameters, we observed changes in the model. Specifically, as the weight of the AHP gradually changed, the ranking of the model evaluation results also varied (Fig. 3B). Reasonable variations in ranking were observed through adjustments in the proportion of subjective and objective weights. Notably, the rankings of ABACUS-R and 3D CNN increased with a higher proportion of subjective weight. This can be attributed to the significantly longer time required by these two methods with iterative strategies in sequence design, which amplified the weight of the time indicator in objective weight calculations. Conversely, ProteinSolver’s ranking decreased rapidly due to the poor folding performance of its generated sequences, while GPD and PiFold experienced gradual declines for similar reasons.

Overall, our model demonstrated stability, with rankings that did not drastically change due to variations in parameters. The observed performance when the subjective weight was too low or too high further affirmed the validity of combining subjective and objective weights, as both prove essential. The results of sensitivity analysis suggested that an AHP weight ratio between 0.4 and 0.6 is reasonable. Therefore, we chose 0.5 as the final proportion of subjective weight within the model. The relatively stable rankings indicate that our model exhibits good generality and stability.

### 3.4 Characteristics of sequence design method

In addition to evaluating various protein design methods, our model aims to provide optimization recommendations. To achieve this, our evaluation model generates normalized and weighted outcomes that can be visualized through a heat map. This allows us to observe the rationality of the weights assigned to different factors and provides insights for optimizing sequence design methods.

**Figure 4.**
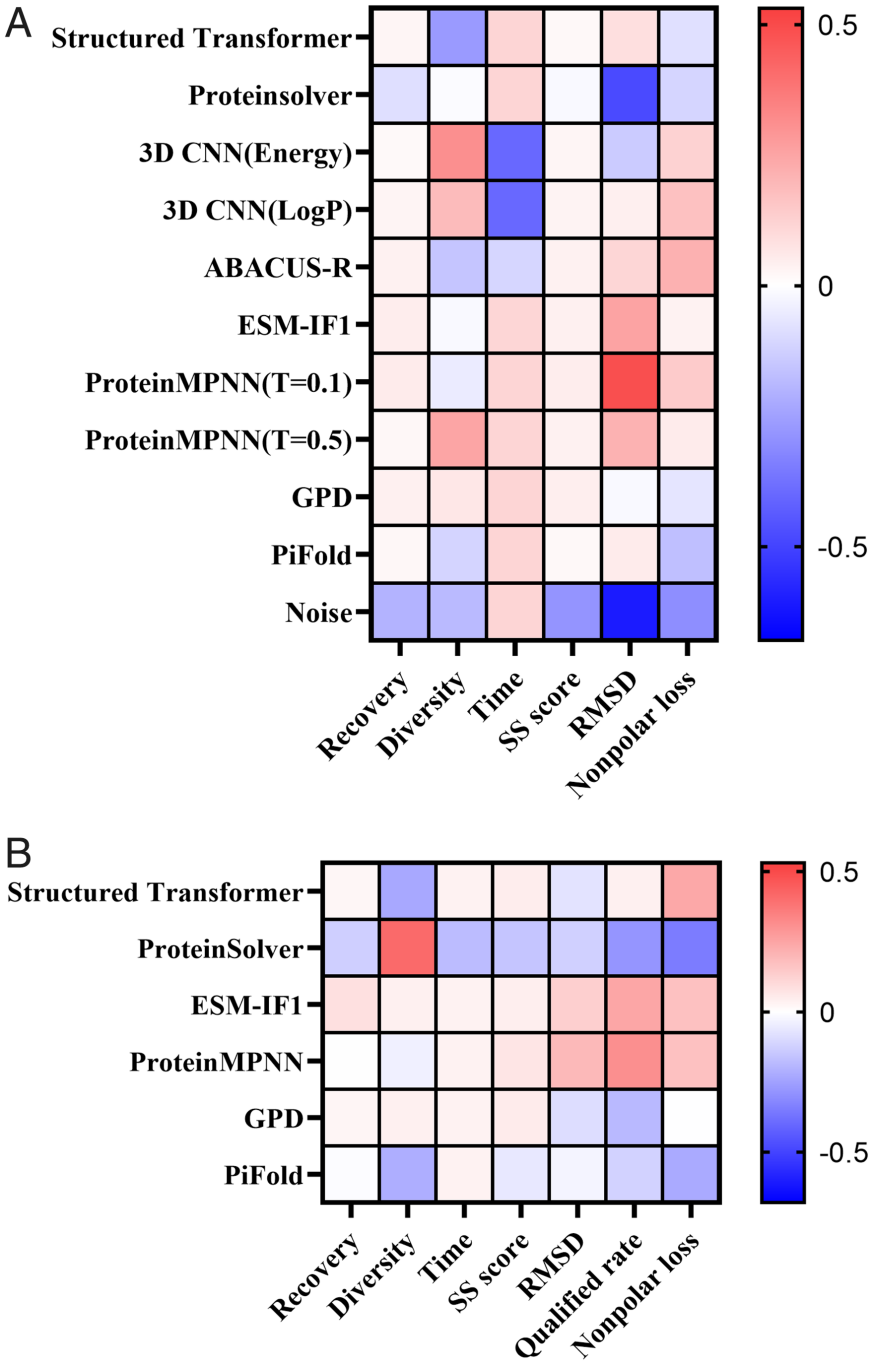
Double gradient heat map of weighted values. (**A**) *De novo* proteins results. For the weighted values of each object, red represents the dominant value and blue represents the inferior value. The advantages and disadvantages of the model can be judged by comparing the same method with different indicators or the same indicators with different methods. (**B**) Single-chain proteins results. The drawing standard is the same as that of *de novo* protein. Because there is no noise method in the single-chain protein sequence design object, the same coloring effect is used.

Among the evaluated methods, the results obtained from the iterative strategy method exhibit a more reasonable protein structure. The superior performance of ProteinMPNN in terms of RMSD is a key factor contributing to its top ranking. Conversely, the rankings of the Structured Transformer method are mainly affected by low diversity, while the 3D CNN method is hampered by the longer computation times required (Fig. 4). Furthermore, PiFold is significantly influenced by a higher Nonpolar loss, indicating a suboptimal folding performance for nonpolar residues. Meanwhile, GPD shows a slightly inferior performance in terms of RMSD. These observations provide valuable insights into the strengths and weaknesses of each method, thus enabling us to make informed recommendations for optimizing sequence design methods.

### 3.5 Continuous repeated amino acid comparison

**Figure 5.**
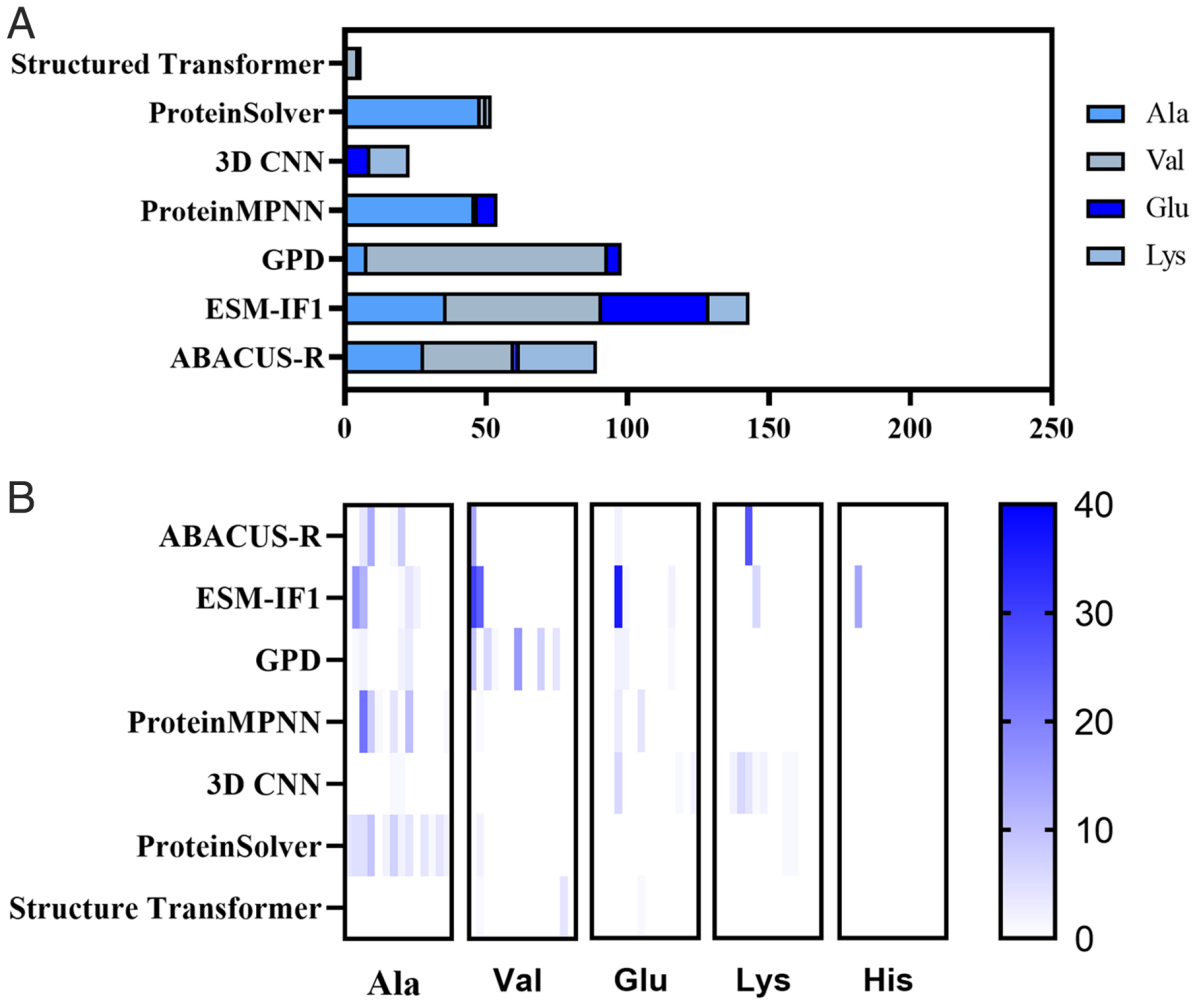
Statistical results of continuous repeated amino acids. (**A**) Stacked Bar Chart of Continuous repeated amino acids. It shows the number of consecutive repeated amino acids in 1400 sequences designed by each method, while the stack diagram only counts four amino acids, namely Ala, Val, Glu, and Lys, which exist in all methods. (**B**) Heat map of the total length of each continuous amino acid repeated on various *de novo* proteins in each method. Each column represents one of the 14 *de novo* proteins, and each cell represents the total length of the corresponding protein designed by the corresponding method with continuous repeats on the corresponding amino acids.

During our analysis of partial sequences, in the process of analyzing some sequences, we found an interesting phenomenon. Many protein sequence design methods have a tendency to design sequences with continuous repeated identical amino acid. Such sequences are highly unfavorable for protein folding in natural conditions and can lead to the formation of amyloid precipitates in solution. To investigate this further, we recorded the occurrences of consecutive, identical amino acids in the designed sequences for each approach, along with their accompanying secondary structures. We found that this phenomenon was primarily restricted to a select range of amino acids, with a focus on four specific ones: Alanine (Ala), Valine (Val), Glutamic acid (Glu), and Lysine (Lys) (Fig. 5A). Additionally, different methods displayed varying degrees of preference for selecting amino acids with continuous repetition. Although this indicator was not included in our comprehensive evaluation model, it provides additional insights into the rationality of sequence design.

The underlying reason for this phenomenon can be easily understood. Deep learning-based protein sequence design methods possess the ability to recognize the contribution of specific amino acids in forming particular secondary structures, such as the α-helix. Consequently, these methods prioritize certain amino acids, resulting in their repeated presence. For instance, Valine is often associated with β-bridges, while Alanine, Glutamic acid, and Lysine are commonly found in α-helices (Supplementary Table S4). This demonstrates the effectiveness of deep learning in protein sequence design scenarios. However, the absence of a corresponding feature or measure to identify and control this anomaly leads to an increased occurrence of continuous repeated amino acids in the generated sequences.

## 4 Discussion

‘The multi-indicator comprehensive evaluation model has proven to be more effective compared with only using recovery to evaluate’. We incorporated more biological indicators, including recovery used in the original evaluation methods. Additionally, the combination of weighting factors enhanced the comprehensiveness of the evaluation (Chen 2020). The results indicate that ProteinMPNN has achieved the best performance at the temperature parameter of 0.5, although it exhibits lower Recovery and RMSD than at the temperature parameter 0.1. At the same time, the ABACUS-R, which is based on an iterative strategy, is ranked higher than GPD, despite the latter having a higher Recovery. These demonstrates that our evaluation model is more comprehensive due to considering multi-indicators and can effectively highlight the strengths and weaknesses of protein sequence design methods from multiple perspectives. However, our evaluation model is based on calculation, which cannot replace the experimental results. At the same time, our model cannot evaluate the huge structural difference or folding failure caused by single point mutation.

‘The multi-indicator comprehensive evaluation model can provide optimization suggestions’. Although the Structured Transformer is greatly limited by Diversity, it does offer parameters to adjust diversity, which can partially compensate for the loss of folding ability in the design sequence by improving design sequence diversity. For the sequence design methods GPD and PiFold, which have similar architectures to Structured Transformer, the main drawback lies in the foldability of the generated sequences. This defect can potentially be addressed by optimizing these methods with reference to the Structured Transformer. Furthermore, among the methods that adopt a concurrent strategy, only ProteinMPNN possesses a Nonpolar loss that can match the performance of iterative methods. Other concurrent strategy methods could potentially learn from ProteinMPNN’s feature extraction methods or architecture to enhance structural rationality. On the other hand, the efficiency of methods employing an iterative strategy is the biggest limitation, and reducing their running time should be prioritized. Additionally, it’s important to note that the data in Fig. 4 are presented as relative indicator values between the methods. In more complex single-chain protein designs, the overall performance of methods utilizing a concurrent strategy is poor, and there is significant room for improvement in each method.

‘Optimizing the continuous repetition of amino acids can significantly enhance the performance of sequence design’. For instance, in the case of GPD, a majority of these repeated amino acids are Valine (Val). It is well-known that Valine repetitiveness aligns closely with the secondary structure of β-bridges. Therefore, in addition to the general optimization methods discussed in the results, focusing on the design of β-bridges could provide valuable optimization insights. ESMFold and ABACUS-R exhibit a higher occurrence of repeated amino acids, while such repetition is absent in native *de novo* proteins and proteins designed by PiFold. Moreover, although Structured Transformer also displays some instances of continuous amino acid repetition, they are relatively rare. We have proposed a potential solution to address the issue of continuous amino acid repetition. Specifically, during the model training process, after obtaining the raw prediction probability of the 20 amino acids, the Kullback–Leibler divergence between the raw prediction probability and the natural proportions of amino acids can be calculated as a new loss function (Bowman *et al.* 2015), weighted with a smaller weight, in conjunction with the original cross-entropy loss. The new loss encourages the design of amino acids for protein folding that conform more closely to the natural distribution, thereby mitigating the occurrence of consecutive repeated amino acids of the same type. This solution is more direct and simple than adjusting the features and architecture of the model, and we will try it in the future work.

‘Optimizing the selection process of the temperature parameter is potential useful’. While our method for selecting temperature parameters has proven to be effective, there are aspects where improvements can be made. For example, using a more precise indicator such as RMSD instead of Recovery could yield more accurate results, especially when the evaluation model assigns a higher weight to RMSD. Additionally, the two indicators can be weighted (Shen *et al.* 2015), allowing users to have greater flexibility in selecting the temperature. This is represented by the stretching of the coordinate axis in Fig. 3B. Moreover, the study only utilized clustering results for a subset of numerical values to determine thresholds, without considering the practical significance of the indicators. Users have the ability to readjust the optimization based on their desired thresholds. It is important to note that when multiple sample points are located within the optimal region, the decision can be based on calculating the distance to the vertex. Therefore, in the process of selecting the best temperature parameters in the future, these operations are helpful to meet the requirements more.

‘Subjective weight can also possess a relative objectivity’. When ranking protein sequence design methods, we adopt a combined approach that incorporates subjective and objective weights. While we have highlighted the significance of subjective weight and recognized the AHP method as the prevailing MDCM method currently (Kubler *et al.* 2016), there is still divergence in subjective weight evaluation due to varying perspectives. This discrepancy in assigning weights can potentially influence the results. To address this, we have employed the FAHP alternative to the AHP method. Although demonstrating the superiority of FAHP over AHP in the results is challenging, it helps reduce the influence of evaluator on the assigned weight. It is important to note that while changes in subjective weight can alter the results, our comprehensive evaluation method ensures stability, reasonability, and interpretability of the results (Supplementary Tables S5–S7). In our model, users have the flexibility to adjust both the judgment matrix and the proportion of subjective weight. By modifying these parameters, users can more effectively select the most appropriate method for their specific application scenarios.

‘The evaluation model should possess the ability to select sequences’. Our aim is to improve the success rate of wet lab validation and minimize experimental costs by identifying sequences with the highest potential for successful folding through our model. Therefore, we have eliminated the diversity indicator and time indicator, as they do not contribute to sequence selection. In another experiment conducted by our research team (Mu *et al.* 2023), we were tasked with identifying the most promising sequence for proper folding out of approximately 1 million considered sequences. The experimental results indicate that three out of the nine sequences we selected for wet experiments exhibited good folding properties. Our goal is for our evaluation model to successfully identify these three sequences out of the nine.

Our results highlight the challenges in accurately selecting sequences that perform well in experiments (Supplementary Tables S8 and S9). Although our model provides some assistance in sequence selection, there are cases where the recommended sequences cannot be expressed and the sequence that can be expressed ranks low. This is attributed to the use of relatively basic indicators in our evaluation model, which require further optimization to enhance precision. For example, the sequence with the highest ranking in our model cannot be expressed, which is related to the fact that our model only considers simple structural indicators. In fact, the indicators related to structural rationality include not only Nonpolar loss, but also SAP (Solvent Accessible Polar) and Net Charge, etc. However, because the main purpose of our model is not to select a suitable sequence, they are not used in this study. When screening the sequences that are more likely to be expressed for experimental verification, the above indicators can be added as screening conditions. However, we must point out that the evaluation of the calculation method cannot give completely accurate results, and it can still only provide some help to the experimental verification.

‘The average value and proportion of qualified RMSD provide promising avenues for further investigation’. In this study, we specifically focused on evaluating single-chain proteins and divided the RMSD indicator into two components: the average value and the proportion of qualified proteins with RMSD less than 2 Å. We applied this computational strategy to assess *de novo* protein sequence design. However, due to insufficient debugging, the outcomes were not satisfactory as reflected in Supplementary Table S10. We argue that utilizing RMSD less than 2 Å is feasible and offers the potential to capture more information compared to directly calculating the mean RMSD. However, further adjustments are necessary for the indicators derived from this partition. These adjustments might include weighing the initial indicators, examining the correlation between the eligible percentage and variety, and refining the selection procedure for qualified proteins. These areas require careful consideration to enhance the effectiveness and reliability of our evaluation process.

## 5 Conclusion

Based on the weighted improved Technique for Order Preference by Similarity to Ideal Solution (TOPSIS) algorithm, we have developed a comprehensive evaluation model for protein sequence design methods. This model has been applied to rank eight deep learning-based methods for protein sequence design. It is a simple yet practical model that can be of great value to decision-makers in this field. During the development of our evaluation model, we carefully selected appropriate indicators to ensure its effectiveness. The results generated by our model not only provide an assessment of the protein sequence design methods but also offer valuable insights into their strengths, weaknesses, and areas for improvement. Additionally, we have discussed the challenges encountered during the design process of the sequence evaluation results. Moreover, we have devised a method for selecting the optimal diversity parameter and proposed solutions to address the common issue of designing sequences with consecutive repeats of the same amino acids, which is observed in many protein design methods. These solutions aim to enhance the quality of the designed sequences and mitigate the potential negative effects. Through our efforts, we aim to contribute to the advancement of protein sequence design methods and provide decision-makers with useful information to guide their choices.

## Supplementary Material

btae037_Supplementary_Data

## References

[btae037-B1] Abdel-Basset M , Mohamed R. A novel plithogenic TOPSIS-CRITIC model for sustainable supply chain risk management. J Clean Prod 2020;247:119586.

[btae037-B2] Anand N , Eguchi R, Mathews II et al Protein sequence design with a learned potential. Nat Commun 2022;13:746. 35136054 10.1038/s41467-022-28313-9 PMC8826426

[btae037-B3] Baker D. What has *de novo* protein design taught us about protein folding and biophysics? Protein Sci 2019;28:678–83. 30746840 10.1002/pro.3588 PMC6423711

[btae037-B4] Bowman SR , Vilnis L, Vinyals O et al Generating sentences from a continuous space. arXiv, arXiv:1511.06349, 2015, preprint: not peer reviewed.

[btae037-B5] Bryant P , Pozzati G, Elofsson A. Improved prediction of protein-protein interactions using AlphaFold2. Nat Commun 2022;13:1265. 35273146 10.1038/s41467-022-28865-w PMC8913741

[btae037-B6] Castorina LV , Petrenas R, Subr K et al PDBench: evaluating computational methods for protein-sequence design. Bioinformatics 2023;39:btad027. 36637198 10.1093/bioinformatics/btad027 PMC9869650

[btae037-B7] Chen C-H. A novel multi-criteria decision-making model for building material supplier selection based on entropy-AHP weighted TOPSIS. Entropy 2020;22:259. 33286032 10.3390/e22020259 PMC7516705

[btae037-B9] Dauparas J , Anishchenko I, Bennett N et al Robust deep learning–based protein sequence design using ProteinMPNN. Science 2022;378:49–56. 36108050 10.1126/science.add2187 PMC9997061

[btae037-B10] Diakoulaki D , Mavrotas G, Papayannakis L. Determining objective weights in multiple criteria problems: the critic method. Comput Oper Res 1995;22:763–70.

[btae037-B11] Ferruz N , Heinzinger M, Akdel M et al From sequence to function through structure: deep learning for protein design. Comput Struct Biotechnol J 2022;21:238–50. 36544476 10.1016/j.csbj.2022.11.014 PMC9755234

[btae037-B12] Gao Z , Tan C, Li SZ. PiFold: toward effective and efficient protein inverse folding. arXiv, arXiv:2209.12643, 2022, preprint: not peer reviewed.

[btae037-B13] Hsu C , Verkuil R, Liu J et al Learning inverse folding from millions of predicted structures. bioRxiv 2022. 10.1101/2022.04.10.487779.

[btae037-B14] Huang B , Fan T, Wang K et al Accurate and efficient protein sequence design through learning concise local environment of residues. Bioinformatics 2023;39:btad122. 36916746 10.1093/bioinformatics/btad122 PMC10027430

[btae037-B15] Ingraham J , Garg V, Barzilay R et al Generative models for graph-based protein design. In: *Advances in Neural Information Processing Systems, Vancouver, Canada* 2019;32.

[btae037-B16] Korendovych IV , DeGrado WF. *De novo* protein design, a retrospective. Q Rev Biophys 2020;53:e3. 32041676 10.1017/S0033583519000131 PMC7243446

[btae037-B17] Kubler S , Robert J, Derigent W et al A state-of the-art survey & testbed of fuzzy AHP (FAHP) applications. Expert Syst Appl 2016;65:398–422.

[btae037-B18] Larkin MA , Blackshields G, Brown NP et al Clustal W and Clustal X version 2.0. Bioinformatics 2007;23:2947–8. 17846036 10.1093/bioinformatics/btm404

[btae037-B19] Lin Z , Akin H, Rao R et al Evolutionary-scale prediction of atomic-level protein structure with a language model. Science 2023;379:1123–30. 36927031 10.1126/science.ade2574

[btae037-B20] Liu Y , Eckert CM, Earl C. A review of fuzzy AHP methods for decision-making with subjective judgements. Expert Syst Appl 2020;161:113738.

[btae037-B21] Liu Y , Kuhlman B. RosettaDesign server for protein design. Nucleic Acids Res 2006;34:W235–W238. 16845000 10.1093/nar/gkl163 PMC1538902

[btae037-B22] Liu Y , Zhang L, Wang W et al Rotamer-free protein sequence design based on deep learning and self-consistency. Nat Comput Sci 2022;2:451–62. 38177863 10.1038/s43588-022-00273-6

[btae037-B23] Mardani A , Jusoh A, Zavadskas EK. Fuzzy multiple criteria decision-making techniques and applications–two decades review from 1994 to 2014. Expert Syst Appl 2015;42:4126–48.

[btae037-B24] Meng Q , Guo F, Tang J. Improved structure-related prediction for insufficient homologous proteins using MSA enhancement and pre-trained language model. Brief Bioinform 2023;24:bbad217. 37321965 10.1093/bib/bbad217

[btae037-B25] Mu J , Li Z, Zhang B et al *De novo* protein sequence design based on deep learning and validation on CalB Hydrolase. bioRxiv 2023, 10.1101/2023.08.01.551444.

[btae037-B26] Qi Y , Zhang JZH. DenseCPD: improving the accuracy of neural-network-based computational protein sequence design with DenseNet. J Chem Inf Model 2020;60:1245–52. 32126171 10.1021/acs.jcim.0c00043

[btae037-B27] Rocklin GJ , Chidyausiku TM, Goreshnik I et al Global analysis of protein folding using massively parallel design, synthesis, and testing. Science 2017;357:168–75. 28706065 10.1126/science.aan0693 PMC5568797

[btae037-B28] Saaty TL. The Analytic Hierarchy Process: Planning, Priority, Setting, Resource Allocation. Pittsburgh, PA: RWS, 1990 .

[btae037-B29] Saltelli A , Ratto M, Andres T et al Global Sensitivity Analysis: The Primer. San Francisco, CA: John Wiley & Sons, 2008.

[btae037-B30] Shen L , Zhou J, Skitmore M et al Application of a hybrid Entropy–McKinsey Matrix method in evaluating sustainable urbanization: a China case study. Cities 2015;42:186–94.

[btae037-B31] Strokach A , Becerra D, Corbi-Verge C et al Fast and flexible protein design using deep graph neural networks. Cell Syst 2020;11:402–411.e4. 32971019 10.1016/j.cels.2020.08.016

[btae037-B32] Tzeng G-H , Huang J-J. Multiple Attribute Decision Making: Methods and Applications. Boca Raton, FL: CRC Press, 2011.

[btae037-B8] UniProt Consortium. UniProt: a worldwide hub of protein knowledge. Nucleic Acids Res 2019;47:D506–15. 30395287 10.1093/nar/gky1049 PMC6323992

[btae037-B33] Wang J , Lisanza S, Juergens D et al Scaffolding protein functional sites using deep learning. Science 2022;377:387–94. 35862514 10.1126/science.abn2100 PMC9621694

[btae037-B34] Woolfson DN. A brief history of *de novo* protein design: minimal, rational, and computational. J Mol Biol 2021;433:167160. 34298061 10.1016/j.jmb.2021.167160

[btae037-B35] Yang W , Jiang X. Evaluating sustainable urbanization of resource-based cities based on the Mckinsey matrix: case study in China. J Urban Plann Dev 2018;144:05017020.

[btae037-B36] Ying C , Cai T, Luo S et al Do transformers really perform bad for graph representation? arXiv, arXiv:2106.05234, 2021, preprint: not peer reviewed.

[btae037-B37] Yue Z. An extended TOPSIS for determining weights of decision makers with interval numbers. Knowl Based Syst 2011;24:146–53.

[btae037-B38] Zadeh LA. Fuzzy sets. Inf Control 1965;8:338–53.

