# Supplementary material for: Multi-indicator comparative evaluation for deep learning-based protein sequence design methods
Source: Bioinformatics. 2024 Jan 23;40(2):btae037. doi: 10.1093/bioinformatics/btae037 (PMC10868333; doi:10.1093/bioinformatics/btae037)
Supplement: btae037_Supplementary_Data [file btae037_supplementary_data.docx]

### Supporting Information

### Multi-indicator Comparative Evaluation for Deep Learning-Based Protein Sequence Design Methods

Jinyu Yu, Junxi Mu, Ting Wei*, Hai-Feng Chen^*^

State Key Laboratory of Microbial metabolism, Joint International Research Laboratory of Metabolic & Developmental Sciences, Department of Bioinformatics and Biostatistics, National Experimental Teaching Center for Life Sciences and Biotechnology, School of Life Sciences and Biotechnology, Shanghai Jiao Tong University, Shanghai, 200240, China

***Corresponding Author**

**Hai-Feng Chen (Full Professor)**

State Key Laboratory of Microbial metabolism, Joint International Research Laboratory of Metabolic & Developmental Sciences, Department of Bioinformatics and Biostatistics, National Experimental Teaching Center for Life Sciences and Biotechnology, School of Life Sciences and Biotechnology, Shanghai Jiao Tong University, Shanghai, 200240, China

**Tel**: 86-21-34204073; **Fax**: 86-21-34204073; **Email**: [haifengchen@sjtu.edu.cn；weitinging@sjtu.edu.cn](mailto:haifengchen@sjtu.edu.cn；weitinging@sjtu.edu.cn)

**Notes**

The authors declare that there is no conflict of interest.

#### Supplementary Text

##### Evaluation indicators

##### Sequence Recovery

Currently, recovery is one of the most widely used evaluation indicator, and many methods consider this indicator when evaluating their final performance. Recovery involves comparing the designed sequence with the native sequence, tallying the occurrence of identical residue types at corresponding positions in both sequences, and subsequently dividing this count by the sequence length. Essentially, this indicator gauges the extent to which the designed sequences can replicate the native sequences, offering insights into the models' ability to perform reverse folding into protein structures.

However, as previously mentioned, the ability to fold specific structures is not solely dependent on specific sequences. Frequently, there exist superior alternatives to natural sequences. For a protein sequence design method, which encompasses the task of protein reverse folding, it becomes crucial to understand the intricate relationship between structure and sequence, extending beyond the limitations of the original sequence and its corresponding structure. Consequently, we will incorporate additional indicators to evaluate the model, including an analysis of the folded structure of the designed sequences and an assessment of sequence diversity. By considering these additional indicators, we aim to provide a more comprehensive evaluation of the model's performance.

###### Sequence Diversity

To enhance the diversity of the generated sequences, we aimed to devise indicators that would effectively evaluate this aspect. We explored several approaches, such as comparing the disparities between the sequences we generated and the native sequence, or considering the entropy among the generated sequences. After careful consideration, we ultimately selected a diversity indicator that focuses on measuring the extent of dissimilarity between the generated sequences rather than comparing them solely to the natural sequences. Clustalw2 was used to align the generated sequences. By examining the pairs of sequences and utilizing the alignment results, we calculated the count of dissimilar and non-identical amino acids between them, taking into account the sequence length. Subsequently, we obtained the average dissimilarity to quantitatively represent the diversity of our generated sequences. This carefully chosen diversity indicator offers a valuable means of assessing the variety within the generated sequences and ensures a more comprehensive evaluation of our model.

After conducting diversity calculations for various methods, we observed that ProteinSolver exhibited a high diversity score but performed poorly in terms of RMSD (see Table S11). This discrepancy indicates that the elevated diversity of ProteinSolver is not attributed to genuinely diverse designed sequences but rather suggests a propensity for inaccurate results. Hence, we propose an enhanced diversity metric as follows:

$Diversity=Diversity\boldsymbol{*}\frac{2}{RMSD}(if RMSD>2)$ (1)

###### Generation Time

We examined time consumption for different methods to generate 100 sequences of the same protein on a CPU. Specifically, we focused on a single-chain protein comprising 261 residues. While most methods exhibited rapid design speeds, a few methods required longer durations due to their iterative algorithms. In our design task, as long as the generation process was completed within a specific computation time, we deemed it acceptable. Rather than solely prioritizing design speed, our primary interest lay in producing superior sequences within a reasonable time frame. To this end, we established a threshold for the completion of 100 sequence generations within 5 minutes. Any method that finished the design process within this time limit was recorded as 5 minutes in our subsequent data analysis. For methods that exceeded the 5-minute mark, we considered their original completion times for further calculations. By imposing this threshold, we aimed to maintain a fair evaluation of the methods based on comparable time frames.

RMSD

The aforementioned indicators primarily focus on the sequence level. However, in order to assess the ability of protein design methods to learn from structures, it is crucial to incorporate structural-level indicators into our evaluation framework.

To achieve this, we employed ESMFold to predict the structures of the 100 designed sequences. Subsequently, we calculated the root mean square deviation (RMSD) between the predicted structures and the original structures. RMSD is a quantitative and fundamental structural evaluation indicator that measures the disparities between two structures. It is extensively used in structural biology to assess the accuracy and reliability of molecular simulations by comparing 3D protein structures.

The RMSD calculation involves determining the Euclidean distance between corresponding atoms in the two structures, squaring the distances of all atoms, summing them, dividing by the number of atoms, and finally taking the square root to obtain the RMSD value. Smaller RMSD values indicate greater similarity between structures. In general, an RMSD value below 2Å is considered indicative of substantial structural similarity between the two evaluated structures.

$RMSD=\sqrt{\frac{1}{N}\sum_{i=1}^{N} {(r_{i}-r_{i}^{'})}^{2}}$ (2)

Where $r_{i}$ represents the coordinates of the atom $i$ in the first structure, $r_{i}^{'}$ represents the coordinates of the atom $i$ in the second structure, and $N$ represents the number of atoms.

###### Nonpolar loss

A functional protein structure should have certain characteristics in terms of its amino acid composition. For example, for proteins that function in solution, their core regions should be non-polar while their surface regions should be polar, while the opposite is true for membrane proteins. Therefore, the recovery of the core region of the solution protein will be higher because the core region is more conserved. However, many methods often overlook this feature and sometimes place non-polar residues on the protein surface, which might be because some training samples come from single chains in protein complexes. We hope to have an indicator that can evaluate whether the structures designed by protein design methods match the structural features found in nature. Therefore, based on existing research, we designed Nonpolar loss to reflect the rationality of amino acid types in the structure. To determine the position of residues, we used the following formula to calculate the number of neighbors of residues within a certain spatial range, which determines whether the residue is located on the surface or in the core.

$n_{i}=\sum_{j=1}^{L} 1/(1+exp(d_{ij}-m))*{((cos(\pi-\Phi_{ij})+a)/(1+a))}^{b}$ (3)

This formula is based on the side-chain neighbor selector in RosettaScripts. The number of neighbors $n_{i}$ for each residue $i$ is calculated as the sum of the weighted contributions of all other residues $j$, where $d_{ij}$ and $\Phi_{ij}$ are the Cb-Cb distance and Ca-Cb / Ca-Cb angle between residue $i$ and residue $j$, respectively. $m=1$, $a=0.5$, and $b=2$ are tuning parameters set as default in RosettaScripts (https://www.rosettacommons.org/docs/latest/scripting_documentation/RosettaScripts/ResidueSelectors/ResidueSelectors).

Because we initially wanted to evaluate the reasonable distribution of hydrophilic and hydrophobic amino acids in the structure, we designed Polar score:

${Polar score=\sum_{i=1}^{L} \theta_{i}*[1-sigmoid(n_{i}-n_{0})]}/{\sum_{i=1}^{L} [1-sigmoid(n_{i}-n_{0})]+}$

${\sum_{i=1}^{L} \delta_{i}*sigmoid(n_{i}-n_{0})}/{\sum_{i=1}^{L} sigmoid(n_{i}-n_{0})}$ (4)

where $L$ is the sequence length; $\delta_{i}$*=1* if residue $i$ is non-polar and *0* otherwise, $\theta_{i}$*=1* if residue $i$ is polar and *0* otherwise, and $n_{0}$ is the median of all $n_{i}$ for residues. $1-sigmoid\left( n_{i}-n_{0} \right)$ ranges from 0 to 1 and is higher when a residue is closer to the surface. It should be noted that some amino acids have weak electrical properties. Therefore, in our definition, serine, threonine, tyrosine, asparagine, and glutamine are considered polar amino acids, while isoleucine, leucine, methionine, tryptophan, phenylalanine, and valine are considered non-polar amino acids.

But the original indicator Polar score (4) had achieved abnormal results on noise method. This is probably because Polar score is a reward-based indicator, and more frequently occurring polar or non-polar amino acids in random sequences results in higher Polar score. In addition, it was worth noting that polar amino acids in the core region of a protein had little effect on protein folding, while non-polar amino acids on the surface region would disrupt protein folding. So we optimized our original indicators and got Nonpolar loss:

$Nonpolar loss{=\sum_{i=1}^{L} \delta_{i}*[1-sigmoid(n_{i}-n_{0})]}/{\sum_{i=1}^{L} [1-sigmoid(n_{i}-n_{0})]}$ (5)

$\delta_{i}$*=1* if residue $i$ is non-polar and *0* otherwise. $1-sigmoid\left( n_{i}-n_{0} \right)$ ranges from 0 to 1 and is higher when a residue is closer to the surface. When there are more non-polar amino acids on the surface, the Nonpolar loss will be higher. After obtaining the Nonpolar loss of the designed structures, we compared it with the results of the original structures to eliminate the influence of structural differences on the Nonpolar loss. It was important to note that our Nonpolar loss here was an evaluation for proteins in solution and did not involve the evaluation of membrane proteins.

###### Secondary Structure score (SS score)

The obtained protein structure represents the tertiary structure, which encompasses the corresponding secondary structures. To assess the model's capacity to learn the structure, we compare the performance of different models in recovering the secondary structures. Although there are similarities to RMSD, the focus here differs. While RMSD emphasizes the overall structure, secondary structure analysis prioritizes specific regions of the structure.

To derive the secondary structure from the tertiary structure, we utilized DSSP. Additionally, we employed a secondary structure prediction tool to anticipate the secondary structure based on the sequence. After conducting a statistical analysis of both methods (Table S12), we ultimately selected PSIPRED for sequence-level secondary structure prediction. We calculated the similarity between the predicted secondary structure of the generated sequences and the native sequence, resulting in the SS score.

The SS score evaluates how effectively the model captures the secondary structure patterns during the protein design process. By comparing the SS scores among different models, we can assess their respective abilities to learn and replicate the desired secondary structure features.

**Evaluation Method**

CRITIC Weight

The CRITIC method uses the standard deviation $S_{j}$ to indicate the variation of values within each indicator. A larger standard deviation indicates a greater variation in protein design methods, which reflects more information and stronger evaluation intensity for that indicator, and therefore it should be assigned a higher weight.

$\left\{ \begin{aligned} \bar{x}_{j}=\frac{1}{n}\sum_{i=1}^{n} x_{ij} \\ S_{j}=\sqrt{\frac{{\sum_{i=1}^{n} (x_{ij}-\bar{x}_{j})}^{2}}{n-1}} \end{aligned} \right.$ (6)

Where $x_{\mathrm{ij}}$ represents the value of the evaluation indicator $j$ of the method $i$. $S_{j}$ is the standard deviation of the indicator $j$. $n$ is the number of methods.

$R_{j}$ is used to represent the correlation between indicators. The higher the correlation with other indicators, the lower the conflict between the indicator and other indicators. This indicated a greater similarity in the information reflected and a higher degree of repetition in the evaluation content. To some extent, this weakened the evaluation strength of the indicator and the weight given to it should be reduced.

$R_{j}=\sum_{i=1}^{P} (1-r_{ij})$ (7)

$r_{ij}$ is the correlation parameter between the evaluation indicators i and j. $P$ is the number of indicators.

$I_{j}=S_{j}\sum_{i=1}^{P} (1-r_{ij})=S_{j}\times R_{j}$ (8)

$I_{j}$ represents the amount of information. The larger $I_{j}$, the greater the role of the evaluation indicator $j$in the whole evaluation indicator system, and it should be given more weight.

Based on the above, the objective weight $w_{j}$ of the indicator $j$ is determined as follows:

$w_{j}=\frac{I_{j}}{\sum_{j-1}^{P} I_{j}}$ (9)

We can get the objective weight of each indicator $W^{1}$.

FAHP Weight

AHP judgment matrix comes from people's subjective judgment, which may come from the summary of objective laws, collective decision-making or expert opinions. The FAHP judgment matrix is essentially the same. When establishing the FAHP judgment matrix, Triangular fuzzy number (TFN) is the most popular means of judgment representation, TFN can be expressed as a triple $(l,m,h)$ where $l$ and $h$ are the smallest and the largest values with the smallest membership respectively and $m$ is the value with the largest membership. For example, in TFN number $\left( 2,3,4 \right)$ $l=2$, $h=4$, $m=3.$ And in this FAHP scenario, $m$ in TFN number represents the importance of this indicator, and $l$ and $h$ are the lower and upper boundaries of this importance. Tables S13-S14 display the fuzzy scales and fuzzy pairwise judgment matrix, respectively.

Then the consistency test of the judgment matrix is carried out. Calculate the index $CI$ that measures the degree of inconsistency of a judgment matrix $A$ (n>1 order square matrix).

$CI=\frac{\lambda_{max}(A)-n}{n-1}$ (10)

Where $\lambda_{max}(A)$ is the maximum eigenvalue of the judgment matrix and n is the order of the judgment matrix. The standard $RI$ for testing the consistency of pairwise judgment matrix $A$, called the average random consistency index, is then checked from relevant information, and it is only relative to the matrix order n. For a matrix order 6, the $RI$ value is 1.24.

The stochastic consistency ratio $CR$ of pairwise judgment matrix $A$ is calculated according to the following formula.

$CR=\frac{CI}{RI}$ (11)

The judgment method is as follows: when $CR$< 0.1, it is judged that the pairwise judgment matrix $A$ has satisfactory consistency or its degree of inconsistency is acceptable; otherwise, the pairwise judgment matrix $A$ is adjusted until satisfactory consistency is achieved. The $CR$ value of our judgment matrix $A$ is 0.07, which meets the condition.

According to Buckley to defuzzify the fuzzy weights, we got the geometric average $r_{i}$ of fuzzy comparison values of each indicator:

$r_{i}=\left( \prod_{j=1}^{n} d_{ij} \right)^{\frac{1}{n}},i=1,2,\ldots,n$ (12)

$d_{ij}$ is the preference value of the indicator $i$ to the indicator $j$ given by the decision maker in the judgment matrix. And the fuzzy weight of each indicator is calculated.

$w_{i}=r_{i}\bigotimes\left( r_{1}\bigoplus r_{2}\bigoplus\ldots\bigoplus r_{n} \right)^{-1}=\left( lw_{i},mw_{i},hw_{i} \right)$ (13)

Using centroid method to defuzzify a TFN:

$w_{i}=\frac{lw_{i}+mw_{i}+hw_{i}}{3}$ (14)

TOPSIS

The ideal best solution represents the optimal values of all indicators in the evaluation system, while the worst solution represents the worst values of all indicators in the evaluation system. To solve the positive ideal solution $S^{+}$ and negative ideal solution $S^{-}$ for TOPSIS, we used the weight matrix $W$ obtained earlier. We multiplied the matrix$X$ by $W$ to obtain the matrix $V$, as shown below:

$V=\left[ \begin{matrix} V_{11} & V_{12} & \ldots& V_{1n} \\ V_{21} & V_{22} & \ldots& V_{2n} \\ \ldots& \ldots& \ldots& \ldots\\ V_{m1} & V_{m2} & \ldots& V_{mn} \end{matrix} \right]=\left[ \begin{matrix} X_{11} & X_{12} & \ldots& X_{1n} \\ X_{21} & X_{22} & \ldots& X_{2n} \\ \ldots& \ldots& \ldots& \ldots\\ X_{m1} & X_{m2} & \ldots& X_{mn} \end{matrix} \right]\times\left[ \begin{matrix} W_{1} & 0 & 0 & 0 \\ 0 & W_{2} & 0 & 0 \\ 0 & 0 & \ldots& 0 \\ 0 & 0 & 0 & W_{n} \end{matrix} \right]$ (15)

At this point

$\left\{ \begin{aligned} S^{+}=max\left\{ V_{ij} | 1\leq i\leq m \right\} \\ S^{-}=min\left\{ V_{ij} | 1\leq i\leq m \right\} \end{aligned} \right.$ (16)

The distance was calculated from the method $i$ to the positive and negative ideal solutions, respectively, where:

$\left\{ \begin{aligned} D_{i}^{+}=\sqrt{\sum_{j=1}^{n} {(V_{ij}-S^{+})}^{2}} \\ D_{i}^{-}=\sqrt{\sum_{j=1}^{n} {(V_{ij}-S^{-})}^{2}} \end{aligned} \right.$ (17)

Next, we calculated the relative nearness of each solution:

$C_{i}=\frac{D_{i}^{-}}{D_{i}^{-}+D_{i}^{+}}$ (18)

Based on the relative nearness, we can rank the solutions, where larger values indicate better design methods.

**Supplementary Table**

Table S1. *De novo* protein and single-stranded protein used in the evaluation process.

| ***De novo* Proteins** | **Protein length** | **Single-chain**  **Proteins** | **Protein length** |
| --- | --- | --- | --- |
| EEHEE_rd3_1498 | **43** | 1nhoA | 85 |
| EEHEE_rd3_0037 | **43** | 1qklA | 127 |
| EHEE_rd1_0882 | **40** | 1v9vA | 104 |
| EHEE_rd2_0005 | **40** | 1xq8A | 140 |
| HEEH_rd2_0779 | **43** | 2a2pA | 130 |
| HEEH_rd3_0223 | **43** | 2a4hA | 148 |
| HEEH_rd3_0726 | **43** | 2b5xA | 148 |
| HHH_rd1_0142 | **43** | 2datA | 123 |
| HHH_rd2_0134 | **43** | 2krxA | 82 |
| EHEE_rd3_0015 | **40** | 2mzbA | 196 |
| HEEH_rd3_0872 | **43** | 2ys8A | 90 |
| EEHEE_rd3_1702 | **43** | 2dj0A | 130 |
| EEHEE_rd3_1716 | **43** | 2k54A | 123 |
| HHH_rd3_0138 | **43** | 2d9eA | 121 |

Table S2. The ranking difference of evaluation model after deleting the best performance method and the worst performance method.

| **Method** | $\boldsymbol{C}_{\boldsymbol{i}}$ | **Rank** | $\mathbf{C}_{\mathbf{i}}$**（Noise）** | **Rank** | $\boldsymbol{C}_{\boldsymbol{i}}$**（Without Noise）** | **Rank** |
| --- | --- | --- | --- | --- | --- | --- |
| Structured Transformer | 0.55748 | 8 | 0.55967 | 6(8) | 0.34185 | 6(8) |
| ProteinSolver | 0.39443 | 10 |  |  |  |  |
| 3D CNN(Energy) | 0.53895 | 9 | 0.5285 | 7(9) | 0.37992 | 5(7) |
| 3D CNN(LogP) | 0.59185 | 6 | 0.58827 | 4(6) | 0.4551 | 3(5) |
| ABACUS-R | 0.61522 | 4 | 0.61176 | 2(4) | 0.4698 | 2(4) |
| ESM-IF1 | 0.72387 | 3 |  |  |  |  |
| ProteinMPNN(T=0.1) | 0.78398 | 2 | 0.78963 | 1(1) | 0.76642 | 1(1) |
| ProteinMPNN(T=0.5) | 0.79183 | 1 |  |  |  |  |
| GPD | 0.61113 | 5 | 0.60439 | 3(5) | 0.39711 | 4(6) |
| PiFold | 0.5661 | 7 | 0.56864 | 5(7) | 0.32558 | 7(9) |
| Noise | 0.28032 | 11 | 0.27111 | 8(11) |  |  |

Table S3. FAHP judgment matrix used in single-chain protein evaluation (TFN is represented by saaty grade).

| **JM sc** | **Recovery** | **Diversity** | **Time** | **SS score** | **Nonpolar loss** | **RMSD** | **Qualified rate** |
| --- | --- | --- | --- | --- | --- | --- | --- |
| Recovery | 1 | 1/3 | 2 | 1/3 | 1/5 | 1/7 | 1/7 |
| Diversity | 3 | 1 | 5 | 3 | 1 | 1/5 | 1/5 |
| Time | 1/2 | 1/5 | 1 | 1/3 | 1/5 | 1/7 | 1/7 |
| SS score | 3 | 1/3 | 3 | 1 | 1/3 | 1/5 | 1/5 |
| Nonpolar loss | 5 | 1 | 5 | 3 | 1 | 1/5 | 1/5 |
| RMSD | 7 | 5 | 7 | 5 | 5 | 1 | 1 |
| Qualified rate | 7 | 5 | 7 | 5 | 5 | 1 | 1 |

Table S4. Detailed statistical results of continuously repeated amino acids and their corresponding secondary structures.

| **Method** | **Ala** | **Val** | **Glu** | **Lys** | **Leu** | **Thr** | **His** |
| --- | --- | --- | --- | --- | --- | --- | --- |
| ABACUS-R | 4(25)^1^  5(1)  6(2) | 4(14)  5(16)  6(2) | 4(2) | 4(27) |  |  |  |
| GPD | 4(6)  5(2) | 4(85) | 4(5) |  |  |  |  |
| Structured Transformer |  | 4(5) | 5(1) |  |  |  |  |
| ProteinSolver | 4(33)  5(11)  6(1)  7(2)  8(1) | 4(2) |  | 4(2) |  |  |  |
| 3D CNN |  |  | 4(9) | 4(13)  5(1) | 4(1) |  |  |
| ProteinMPNN | 4(39)  5(3)  6(3)  8(1) | 4(1) | 4(6)  5(1) |  |  | 4(1) |  |
| ESM-IF1 | 4(23)  5(7)  6(6) | 4(55) | 4(38) | 4(14) |  |  | 4(1)  5(3)  6(3)  7(2)  10(6) |
| DSSP^2^ | H | E(Most)  -(Few) | H(Most)  S(Few) | H(Most)  -(Few) | E | E | E(Part)  H(Part) |

1: 4(25) means that four amino acids are repeated continuously, which appears 25 times in the designed 1400 sequences. 2:Secondary structure corresponding to repeated sequence identify with DSSP. H stands for α-helix, E stands for β-folded sheet,- stands for random curl, and S stands for high curvature secondary structure.

Table S5. Another FAHP judgment matrix JM2 used in *de novo* protein evaluation (TFN is represented by saaty grade).

| **JM2** | **Recovery** | **Diversity** | **Time** | **SS score** | **RMSD** | **Nonpolar loss** |
| --- | --- | --- | --- | --- | --- | --- |
| Recovery | 1 | 1 | 3 | 1/3 | 1/7 | 1/3 |
| Diversity | 1 | 1 | 3 | 1/3 | 1/7 | 1/3 |
| Time | 1/3 | 1/3 | 1 | 1/5 | 1/7 | 1/5 |
| SS score | 3 | 3 | 5 | 1 | 1/5 | 3 |
| RMSD | 7 | 7 | 7 | 5 | 1 | 7 |
| Nonpolar loss | 3 | 3 | 5 | 1/3 | 1/7 | 1 |

Table S6. The multiple judgment matrix. JM1 and JM 2 (Table S8) are aggregated using geometric average method.

| **Multiple JM(MJM)** | **Recovery** | **Diversity** | **Time** | **SS score** | **RMSD** | **Nonpolar loss** |
| --- | --- | --- | --- | --- | --- | --- |
| Recovery | (1,1,1) | (0.5,0.577,0.707) | (1.414,2.449,3.464) | (0.25,0.333,0.5) | (0.125,0.143,0.167) | (0.204,0.258,0.354) |
| Diversity | (1.414,1.732,2) | (1,1,1) | (2.828, 3.873, 4.899) | (0.707,1,1.414) | (0.144,0.169,0.204) | (0.5,0.577,0.707) |
| Time | (0.289,0.408,0.707) | (0.204,0.258,0.354) | (1,1,1) | (0.204,0.258,0.354) | (0.125,0.143,0.167) | (0.167,0.2,0.25) |
| SS score | (2,3,4) | (0.707,1,1.414) | (2.828,3.873,4.899) | (1,1,1) | (0.167,0.2,0.25) | (0.5,0.577,0.707) |
| RMSD | (6,7,8) | (4.899,5.916,6.928) | (6,7,8) | (4,5,6) | (1,1,1) | (4,5,6) |
| Nonpolar loss | (2.828,3.873,4.899) | (1.414,1.732,2) | (4,5,6) | (1.414,1.732,2) | (0.167,0.2,0.25) | (1,1,1) |

Table S7. Evaluation results of AHP and FAHP when using different evaluation matrices JM1 and MJM (Table S9).

| **METHOD** | **Fuzzy AHP JM1** | **Rank1** | **Fuzzy AHP MJM** | **Rank2** | **AHP JM1** | **Rank3** | **AHP MJM** | **Rank4** |
| --- | --- | --- | --- | --- | --- | --- | --- | --- |
| Structured  Transformer | 0.557 | 8 | 0.587 | 7 | 0.557 | 8 | 0.590 | 6 |
| ProteinSolver | 0.394 | 10 | 0.393 | 10 | 0.390 | 10 | 0.387 | 10 |
| 3D CNN  (Energy) | 0.539 | 9 | 0.528 | 9 | 0.538 | 9 | 0.523 | 9 |
| 3D CNN(LogP) | 0.592 | 6 | 0.591 | 6 | 0.592 | 6 | 0.588 | 8 |
| ABACUS-R | 0.615 | 4 | 0.639 | 4 | 0.615 | 4 | 0.639 | 4 |
| ESM-IF1 | 0.724 | 3 | 0.745 | 3 | 0.724 | 3 | 0.747 | 3 |
| ProteinMPNN  (T=0.1) | 0.784 | 2 | 0.814 | 1 | 0.785 | 2 | 0.818 | 1 |
| ProteinMPNN  (T=0.5) | 0.792 | 1 | 0.790 | 2 | 0.791 | 1 | 0.789 | 2 |
| GPD | 0.611 | 5 | 0.617 | 5 | 0.609 | 5 | 0.616 | 5 |
| PiFold | 0.566 | 7 | 0.586 | 8 | 0.566 | 7 | 0.589 | 7 |
| Noise | 0.280 | 11 | 0.276 | 11 | 0.277 | 11 | 0.274 | 11 |

The change of JM matrix will only lead to a small range of ranking changes, and the change of FAHP will be smaller than that of AHP.

Table S8. FAHP judgment matrix used in *de novo* sequence selection (TFN is represented by saaty grade).

| **JM select** | **Recovery** | **SS score** | **RMSD** | **Nonpolar loss** |
| --- | --- | --- | --- | --- |
| Recovery | 1 | 1/3 | 1/7 | 1/5 |
| SS score | 3 | 1 | 1/5 | 1/3 |
| RMSD | 7 | 5 | 1 | 5 |
| Nonpolar loss | 5 | 3 | 1/5 | 1 |

Table S9. For the selection results of nine experimental sequences, the sequence design263, design323 and design2422 are the actually selected sequence. FAHP judgment matrix are list in Table S12.

| **Seq** | **Recovery** | **SS score** | **RMSD(Å)** | **Nonpolar loss** | $\mathbf{C}_{\mathbf{i}}$ | **Rank** | **GBSA(kcal/mol)** | **Expressed** | **Soluble** | **Activity(%)** |
| --- | --- | --- | --- | --- | --- | --- | --- | --- | --- | --- |
| design263 | 0.498 | 0.849 | 2.461 | 0.904 | 0.66542 | 2 | -45.03 | √ | √ | 36.14 |
| design323 | 0.495 | 0.855 | 2.807 | 0.922 | 0.47959 | 4 | -44.14 | √ | √ | 68.76 |
| design797 | 0.489 | 0.834 | 3.225 | 0.813 | 0.42154 | 5 | -42.31 |  |  |  |
| design1618 | 0.483 | 0.826 | 3.285 | 0.826 | 0.3716 | 7 | -46.9 |  |  |  |
| design2422 | 0.479 | 0.826 | 2.551 | 0.950 | 0.49739 | 3 | -43.74 | √ | √ | 33.82 |
| design3841 | 0.473 | 0.868 | 3.382 | 0.826 | 0.41208 | 6 | -43.19 | √ | √ | 16.34 |
| design5803 | 0.464 | 0.883 | 2.398 | 0.863 | 0.78069 | 1 | -45.27 |  |  |  |
| design5916 | 0.461 | 0.823 | 3.372 | 0.895 | 0.18398 | 9 | -44.09 | √ | √ | 25.13 |
| design7774 | 0.445 | 0.864 | 3.296 | 0.950 | 0.23525 | 8 | -44.51 |  |  |  |
| WT |  |  |  |  |  |  | -42.41 |  |  |  |

**GBSA means The GBSA(Generalized Born Surface Area) binding free energy**

Table S10. Evaluation results of *de novo* sequence design using qualified RMSD and its proportion.

| **METHOD** | **Recovery** | **Diversity** | **Time** | **SS score** | **Nonpolar loss** | **RMSD(Å)** | **Qualified R**[**ate**](https://fanyi.sogou.com/javascript:%20void(0)) | $\boldsymbol{C}_{\boldsymbol{i}}$ | **Rank** |
| --- | --- | --- | --- | --- | --- | --- | --- | --- | --- |
| 3D CNN(LogP) | 0.445 | 0.272 | 536544 | 0.952 | 1.027 | 0.828 | 0.620 | 0.679 | 4 |
| ABACUS-R | 0.457 | 0.124 | 233280 | 0.972 | 0.968 | 0.866 | 0.606 | 0.636 | 6 |
| Structured Transformer | 0.441 | 0.074 | 180 | 0.966 | 1.352 | 0.871 | 0.571 | 0.572 | 8 |
| ESM-IF1 | 0.477 | 0.184 | 13 | 0.965 | 1.201 | 0.827 | 0.615 | 0.688 | 3 |
| ProteinMPNN  (T=0.1) | 0.487 | 0.168 | 1980 | 0.975 | 1.061 | 0.782 | 0.661 | 0.724 | 2 |
| GPD | 0.462 | 0.219 | 112 | 0.967 | 1.333 | 1.053 | 0.439 | 0.471 | 9 |
| PiFold | 0.428 | 0.141 | 35 | 0.945 | 1.464 | 0.862 | 0.584 | 0.589 | 7 |
| 3D CNN(Energy) | 0.421 | 0.346 | 536544 | 0.931 | 1.085 | 0.836 | 0.574 | 0.678 | 5 |
| ProteinSolver | 0.246 | 0.498 | 35 | 0.792 | 1.389 | 1.259 | 0.224 | 0.376 | 10 |
| ProteinMPNN  (T=0.5) | 0.430 | 0.299 | 221 | 0.965 | 1.174 | 0.796 | 0.606 | 0.770 | 1 |

Table S11. The initial statistical results of indicators.

| **METHOD** | **Recovery** | **Diversity** | **Time（s）** | **SS score** | **RMSD(Å)** | **Polar score** |
| --- | --- | --- | --- | --- | --- | --- |
| 3D CNN(LogP) | 0.445 | 0.272 | 536544 | 0.952 | 1.620 | 0.839 |
| ABACUS-R | 0.457 | 0.124 | 233280 | 0.972 | 1.482 | 0.848 |
| ProteinSolver | 0.246 | 0.498 | 180 | 0.792 | 5.354 | 1.145 |
| Structure Transformer | 0.441 | 0.074 | 13 | 0.966 | 1.526 | 0.943 |
| ESM-IF1 | 0.477 | 0.184 | 1980 | 0.965 | 1.265 | 0.900 |
| ProteinMPNN | 0.487 | 0.168 | 112 | 0.975 | 1.019 | 0.875 |
| GPD | 0.462 | 0.219 | 35 | 0.967 | 1.758 | 0.902 |
| 3D CNN(Energy) | 0.421 | 0.346 | 536544 | 0.931 | 2.130 | 0.945 |
| Noise | 0.049 | 0.656 | - | 0.185 | 11.830 | 1.230 |

Table S12. Evaluation result of SS score calculated by DSSP.

| **METHOD** | **Recovery** | **Diversity** | **Time(s)** | **SS score** | **RMSD** | **Nonpolar loss** | $\mathbf{C}_{\mathbf{i}}$ | **Rank** |
| --- | --- | --- | --- | --- | --- | --- | --- | --- |
| Structured Transformer | 0.441 | 0.074 | 13 | 0.966 | 1.526 | 1.352 | 0.559 | 8 |
| ProteinSolver | 0.246 | 0.186 | 180 | 0.792 | 5.354 | 1.389 | 0.392 | 10 |
| 3D CNN(Energy) | 0.421 | 0.325 | 536544 | 0.931 | 2.130 | 1.085 | 0.540 | 9 |
| 3D CNN(LogP) | 0.445 | 0.272 | 536544 | 0.952 | 1.620 | 1.027 | 0.593 | 6 |
| ABACUS-R | 0.457 | 0.124 | 233280 | 0.972 | 1.482 | 0.968 | 0.615 | 4 |
| ESM-IF1 | 0.477 | 0.184 | 1980 | 0.965 | 1.265 | 1.201 | 0.723 | 3 |
| ProteinMPNN  (T=0.1) | 0.487 | 0.168 | 112 | 0.975 | 1.019 | 1.061 | 0.783 | 2 |
| ProteinMPNN  (T=0.5) | 0.430 | 0.299 | 112 | 0.965 | 1.320 | 1.174 | 0.792 | 1 |
| GPD | 0.462 | 0.219 | 35 | 0.967 | 1.758 | 1.333 | 0.610 | 5 |
| PiFold | 0.428 | 0.141 | 221 | 0.945 | 1.592 | 1.464 | 0.567 | 7 |
| Noise | 0.049 | 0.111 | - | 0.196 | 11.830 | 1.628 | 0.280 | 11 |

Table S13. Fuzzy scales.

| **Saaty grade** | **Language definition** | **TFN** |
| --- | --- | --- |
| 1 | equal importance | (1,1,1) |
| 3 | moderate importance | (2,3,4) |
| 5 | strong importance | (4,5,6) |
| 7 | Very strong importance | (6,7,8) |
| 9 | extremely importance | (9,9,9) |
| 2 4 6 8 | Interval value between two adjacent scales | (1,2,3) (3,4,5) (5,6,7) (7,8,9) |

If indicator $i$ is moderate importance than indicator $j$, For $d_{ij}$ in judgment matrix $A$, $d_{ij}=\left( 2,3,4 \right)$, $d_{ji}=\left( 1/4,1/3,1/2 \right)$.

Table S14. FAHP judgment matrix used in *de novo* protein evaluation

| **JM1 *de novo*** | **Recovery** | **Diversity** | **Time** | **SS score** | **RMSD** | **Nonpolar loss** |
| --- | --- | --- | --- | --- | --- | --- |
| Recovery | 1 | 1/3 | 3 | 1/3 | 1/7 | 1/5 |
| Diversity | 3 | 1 | 5 | 3 | 1/5 | 1 |
| Time | 1/3 | 1/5 | 1 | 1/3 | 1/7 | 1/5 |
| SS score | 3 | 1/3 | 3 | 1 | 1/5 | 1/3 |
| RMSD | 7 | 5 | 7 | 5 | 1 | 5 |
| Nonpolar loss | 5 | 1 | 5 | 3 | 1/5 | 1 |

TFN is represented by saaty grade in Table S13. For example, $3=\left( 2,3,4 \right)$, $1/3=\left( 1/4,1/3,1/2 \right)$.
